# Simultaneous quantification of natural and inducible regulatory T-cell subsets during interferon-β therapy of multiple sclerosis patients

**DOI:** 10.1186/s12967-020-02329-5

**Published:** 2020-04-16

**Authors:** Marco Chiarini, Ruggero Capra, Federico Serana, Diego Bertoli, Alessandra Sottini, Viviana Giustini, Cristina Scarpazza, Marco Rovaris, Valentina Torri Clerici, Diana Ferraro, Simonetta Galgani, Claudio Solaro, Marta Zaffira Conti, Andrea Visconti, Luisa Imberti, SURROGATE Study Group

**Affiliations:** 1 Clinical Chemistry Laboratory, Diagnostic Department, ASST Spedali Civili, Brescia, Italy; 2 Multiple Sclerosis Center, ASST Spedali Civili, Brescia, Italy; 3 Department of Molecular and Translational Medicine, University of Brescia, Brescia, Italy; 4 Centro di Ricerca Emato-oncologica AIL (CREA), ASST Spedali Civili, P.le Spedali Civili 1, 25123 Brescia, Italy; 5 Department of General Psychology, University of Padua, Padova, Italy; 6 IRCCS Fondazione Don Carlo Gnocchi, Milan, Italy; 7 Fondazione IRCCS Istituto Neurologico Carlo Besta, Milan, Italy; 8 Dipartimento di Scienze Biomediche, Metaboliche e Neuroscienze, University of Modena and Reggio Emilia, Modena, Italy; 9 Azienda Ospedaliera San Camillo-Forlanini, Rome, Italy; 10 Department of Rehabilitation, CRRF Mons Luigi Novarese Moncrivello, Vercelli, Italy; 11 Azienda Ospedaliera Papa Giovanni XXIII, Bergamo, Italy; 12 Merck Serono SPA, Rome, Italy

**Keywords:** Multiple sclerosis, Interferon-β, Regulatory T cells, Treg subsets

## Abstract

**Background:**

The mechanisms underlying the therapeutic activity of interferon-β in multiple sclerosis are still not completely understood. In the present study, we evaluated the short and long-term effects of interferon-β treatment on different subsets of regulatory T cells in relapsing–remitting multiple sclerosis patients biologically responsive to treatment because of mixovirus resistance protein A inducibility.

**Methods:**

In this prospective longitudinal study, subsets of natural regulatory T cells (naïve, central memory and effector memory) and inducible regulatory T cells (Tr1), as well as in vitro-induced regulatory T cells (Tr1-like cells), were simultaneously quantified by flow cytometry in samples prepared from 148 therapy-naïve multiple sclerosis patients obtained before and after 6, 12, 18, and 24 months of interferon-β-1a treatment. mRNA for interleukin-10 and Tr1-related genes (CD18, CD49b, and CD46, together with Cyt-1 and Cyt-2 CD46-associated isoforms) were quantified in Tr1-like cells.

**Results:**

Despite profound inter-individual variations in the modulation of all regulatory T-cell subsets, the percentage of natural regulatory T cells increased after 6, 12, and 24 months of interferon-β treatment. This increase was characterized by the expansion of central and effector memory regulatory T-cell subsets. The percentage of Tr1 significantly enhanced at 12 months of therapy and continued to be high at the subsequent evaluation points. Patients experiencing relapses displayed a higher percentage of naïve regulatory T cells and a lower percentage of central memory regulatory T cells and of Tr1 before starting interferon-β therapy. In addition, an increase over time of central memory and of Tr1 was observed only in patients with stable disease. However, in vitro-induced Tr1-like cells, prepared from patients treated for 24 months, produced less amount of interleukin-10 mRNA compared with pre-treatment Tr1-like cells.

**Conclusion:**

Interferon-β induces the expansion of T regulatory subsets endowed with a high suppressive activity, especially in clinically stable patients. The overall concurrent modulation of natural and inducible regulatory T-cell subsets might explain the therapeutic effects of interferon-β in multiple sclerosis patients.

## Background

Multiple sclerosis (MS) is an autoimmune, inflammatory and chronic disease of central nervous system. Interferon-β (IFN-β) is one of the main treatments of the relapsing–remitting form of MS (RRMS); however, its mechanism of action is multifactorial and still under debate. A better understanding of the actions underlying the effects of IFN-β and its anti-inflammatory properties would help to clarify why IFN-β is not effective in all MS patients and to identify subjects that may benefit most from IFN-β treatment and those that should be directed to a therapy switch.

Given the central role in suppressing T-cell activity and inflammation, regulatory T cells (Treg) are potential biomarkers of IFN-β therapeutic efficacy in MS [1]. Treg can be divided into naturally occurring Treg (nTreg) and inducible Treg (iTreg) [2]. FoxP3 is the classic marker of Treg and the presence of the FoxP3 exon2 isoform has proved to pinpoint the CD4^+^ Treg with the highest regulatory activity [2, 3]. Treg can also be identified as CD4^+^CD25^+^ cells with low expression or absence of interleukin-7 receptor alpha chain (IL‐7R or CD127) [4]. Antigen-specific activation in secondary lymphoid organs gives rise to effector memory Treg (TregEM) that migrate to antigen-expressing peripheral tissues, where they stably reside or recirculate between blood and non-lymphoid tissues, and central memory Treg (TregCM) that remain in secondary lymphoid organs [5]. The chemokine receptor CCR7, which regulates cellular trafficking both in lymphoid and inflamed sites, is the marker that distinguishes TregCM and TregEM and is responsible for the ex vivo function of nTreg [5]. The expression of CD120b (also known as tumour necrosis factor receptor 2 or TNFR2) on the cell surface appears to confer to nTreg a maximal suppressive activity [6].

The identification of Tr1, one of the main iTreg subsets, is complex although some reports propose the combined expression of CD18 and CD49b integrins as Tr1 cell-specific markers [7]. Furthermore, it is possible to generate in vitro-induced Tr1 (Tr1-like cells) by stimulating cells with anti-CD3 and anti-CD46 monoclonal antibodies (MoAbs). The anti-CD3 MoAb is used as a general T-cell activation stimulus, while anti-CD46 MoAb is a specific Tr1-like cell inducer [8].

Several researchers have studied the effects of IFN-β treatment either on peripheral nTreg or iTreg [9–15], but no previous study has investigated the modulation of nTreg and their subsets, Tr1 and Tr1-like cells in the same cohort of patients. Most reports on Treg are based on cross-sectional studies and limited by selection bias, such as a small number of included patients and a short follow-up. Finally, most studies missed the detection of anti-IFN-β antibodies affecting the IFN-β bioactivity [16].

Therefore, we performed a long-term study to quantify nTreg and their subsets, Tr1 and Tr1-like cells in a large cohort of therapy-naïve MS patients who initiated IFN-β treatment and were biologically IFN-β responders.

## Methods

### Patients and controls

This prospective longitudinal study enrolled therapy-naïve patients (n = 148) involving 23 MS centres across Italy. Samples from age-matched healthy subjects served as controls (Ctrl) for in vitro studies.

All subjects signed an informed consent form. The study was approved by Ethical Committees of Brescia (protocol n. 28961 of June 21, 2012) and of each MS Center, and was performed in accordance with the specific Italian regulation on observational studies and in compliance with the Italian regulations on non-interventional trials.

The 148 patients were included if they received a confirmed diagnosis of RRMS, according to the 2010 Mc Donald criteria [17]. They were treated with 22 or 44 μg of IFN-β-1a subcutaneous, injected three times per week. Each patient was followed for a 24-months period starting from the first IFN-β administration, except in cases of dropout from the study, who had a shorter follow-up. Blood samples were obtained before the first IFN-β injection (T0) and then at 6 (T6), 12 (T12), 18 (T18), and 24 (T24) months of follow-up. Blood samples at T12 and T24 were also collected in PAXgene tubes (Blood RNA Tube, PreAnalytiX, Hombrechtikon, Switzerland) 11–13 h after the last IFN-β administration, in order to allow for a correct mixovirus resistance protein A (MxA) mRNA induction assessment.

Results were reported according with the STROBE guidelines [18].

### Magnetic resonance imaging examination

Magnetic resonance imaging (MRI) have been performed using a 1.5 T scanner commonly used in clinical routine. T1-weighted images with administration of a single dose of contrast medium (gadolinium-DTPA, 0.1 mmol/kg; i.e. 0.2 ml/kg) as well as T2-weighted images (double-echo MRI brain scans) have been performed according to Italian clinical practice (https://www.ncbi.nlm.nih.gov/pubmed/23828372). Number and volume of gadolinium-enhancing lesions on T1-weighted and new/enlarging lesions on T2-weighted sequences have been centrally measured by MRI experienced readers at the centralized MRI Lab (Fondazione Don Gnocchi Institute, Milan).

### Sample processing

Twenty ml of blood collected in ethylenediaminetetraacetic acid tubes were received by a controlled-temperature next-day shipping at every time point. An aliquot of blood underwent flow cytometry immediately, while the remaining blood underwent standard Ficoll density gradient centrifugation in order to obtain peripheral blood mononuclear cells (PBMC), which were then stored in liquid nitrogen in a solution containing 90% fetal calf serum (FCS) and 10% dimethylsulfoxide until the in vitro experiments. Blood for MxA mRNA quantification was stored at − 20 °C for up to 6 months.

### Treg subset phenotyping

T-cell subsets were assessed by 8-colour flow cytometry analysis on 150 μl whole blood. Cells were stained with various combinations of optimal staining concentrations (previously determined by titrations) of horizon V500 anti-CD4, APC-H7 anti-CD45RA, BV421 anti-CD25, PerCP-Cy5-5 anti-CCR7, PE-Cy7 anti-CD127, anti-PE CD120b, FITC anti-CD49b, and APC anti-CD18 MoAbs (BD Pharmingen, S. Diego, CA; Biolegend, S. Diego, CA and eBioscience, Vienna, Austria).

The nTreg population was identified as CD4^+^CD25^+^CD127^low/‒^ cells. By using the anti-CD45RA and anti-CCR7 antibodies, the following nTreg subsets were identified: naïve Treg CD4^+^CD25^+^CD127^low/‒^CD45RA^+^CCR7^+^, TregCM CD4^+^CD25^+^CD127^low/‒^CD45RA^‒^CCR7^+^, and TregEM CD4^+^CD25^+^CD127^low/‒^CD45RA^‒^CCR7^‒^ [19]. CD4^+^CD25^+^CD127^low/‒^ cells were also characterized for the expression of CD120b marker. The values of all these subsets were expressed as percentage and absolute number.

Similarly, CD4^+^ cells were evaluated for CD18 and CD49b markers to define the Tr1 population as CD4^+^CD18^+^CD49b^+^ lymphocytes [7]. This cell subset was also evaluated before and after in vitro induction of Tr1-like cells, starting from CD4^+^ cells, isolated from PBMC of patients and Ctrl, stimulated with anti-CD3 and anti-CD46 MoAbs in presence of interleukin-2 (IL-2).

Data acquisition was performed with a 3-laser 8-color FACSCanto II cytometer and analyzed with the FACSDiva software (BD Biosciences, San Jose, CA) and FlowJo (FlowJo LLC, Ashland, OR), and results were expressed as percentage of cells or as mean fluorescence intensity (MFI).

### Induction of Tr1-like cells

In vitro induction of Tr1-like cells was performed after thawing about 20 × 10^6^ PBMC of 60 patients obtained before IFN-β therapy initiation and after 12 and 24 months. Patients were randomly chosen among those who completed the T24 follow-up and resulted always MxA-induced. CD4^+^ cells (purity > 95% according to flow cytometer analysis) were prepared using the CD4^+^ T Cell Isolation Kit (Miltenyi Biotec, Gladbach, Germany), according to manufacturer’s protocol. 3 × 10^6^ CD4^+^ cells, diluted in complete RPMI 1640 (2 mM l-glutamine, 100 U/ml penicillin, and 100 µg/ml streptomycin) supplemented with 20% FCS and 40 U/ml of human recombinant IL-2 (Sigma-Aldrich, Saint Louis, MO), were incubated in previously coated 24 well-plates with 10 μg/ml of anti-CD3 (clone OKT3; BioXcell, West Lebanon, NH) and anti-CD46 (clone E4.3; Santa Cruz Biotechnology, Santa Cruz, CA) MoAbs; the MoAbs used for coating were not eliminated, therefore their final concentration was 5 µg/ml. Remaining 3 × 10^6^ CD4^+^ cells were diluted in complete RPMI 1640 supplemented with 20% FCS, without any type of stimulus.

Total RNA for molecular studies was extracted from 2 × 10^6^ of both unstimulated and stimulated CD4^+^ cells for 24 h; 1 × 10^6^ CD4^+^ cells were left in culture for further 24 h for Tr1-like cell quantification by flow cytometry.

### Real-time PCR for quantification of MxA and Tr1-related gene mRNAs

Total RNA was prepared either from whole blood collected into PAXgene tubes using the PAXgene Blood RNA Kit (PreAnalytiX), or extracted from unstimulated and anti-CD3/CD46 MoAb co-stimulated CD4^+^ cells using NucleoSpin RNA II kit (Machery-Nagel, Düren, Germany). RNA to cDNA retrotranscription was performed with Taqman reverse transcription reagents using random hexamers. cDNA for MxA mRNA quantification was subjected to quantitative real-time PCR in a 7500 Fast Real-time PCR system, using primers and probes specific for MxA and the glyceraldehyde 3-phosphate-dehydrogenase as reference gene, as reported [20].

Taqman gene expression assay (Thermo Fisher Scientific, Waltham, MA) were used to analyze the expression of mRNA for the following genes: interleukin-10 (IL-10; Hs00961622_m1, VIC), CD46 (Hs00611256_m1, FAM), CD49b (Hs00158127_m1, VIC), CD18 (Hs00164957_m1, FAM): glucuronidase β gene (GUSB-Hs00939627_m1, FAM) was chosen as the housekeeping reference gene.

The primers and probes for Cyt-1 and Cyt-2 CD46 isoform quantification were the following:

Cyt-1: 5′-GATGAGACCCACAGAGAAGTAAAATTTAC-3′ (forward), 5′-ACCATCTGCTTTCCTTTTAATGATAAA-3′ (reverse), and 5′-FAM-TCTCTCTGAGAAGGAGAGATGAGAGAAAGGTTTGC-TAMRA-3′ (probe).

Cyt-2: 5′-AGAAGAAAGGGAAAGCAGATGGT-3′ (forward), 5′-CAGAGCAGAGAGGCTGAATAGATTC-3' (reverse) and 5′-HEX-GAGCTGAATATGCCACTTACCAGACTAAATCAACCAC-TAMRA-3′ (probe).

The Cyt-1 primers were designed in the Cyt-1-specific exon 13, while the Cyt-2 forward primer overlap the junction between exons 12 and 14. Assays were run on a 7500 Fast Real-time PCR instrument (Life Technologies, Foster City, CA) and data were analyzed with the 7500 Fast System SDS software (Life Technologies) to determine the cycle threshold (Ct). Results were then obtained using a modified version of the comparative “ΔΔ Ct” method and expressed as normalization ratio (NR) that took into account the different amplification efficiencies of the primer set used for the different targets, as suggested by Pfaffl et al. [21].

### Statistical analysis

For the analysis of follow-up data (ex vivo flow cytometry studies), in order to control for any selection bias due to study dropout of patients non responsive to the therapy (or for any non-random loss of participants), an intention to treat analysis was performed using all available samples (i.e. if a subject dropped out from the study, any available samples of previous time points were retained). Therefore, ANOVA for repeated measures using linear mixed models (with fixed and random effects) were employed, as these methods allow analysis even in case of missing samples. Planned contrasts or Bonferroni-corrected post hoc tests were performed to compare subgroups. Correlations were analyzed using the Pearson correlation coefficient. Data of in vitro gene expression studies that did not fulfill normality assumptions were log-converted; if outliers were detected, data were excluded from the analysis. Multivariable regression was fit using linear mixed models, with stepwise backward elimination of covariates. To provide a description of the samples, categorical variables (for instance, gender and enhancing lesion yes/no) are expressed as count (percentage) and continuous variables as mean and standard deviation (SD). Between-group differences were tested using the Chi squared or the two independent sample t- tests for categorical and continuous variables, respectively. For statistical analysis on new relapses and new lesions, after a mixed ANOVA, the Newman-Keuls post hoc test was used.

P-value threshold for significance was set at 0.05. If not otherwise reported, significant p-values in the figures are < 0.05. Data were analyzed using Stata Statistical Software Release 12 (StataCorp LP, College Station, TX) and GraphPad Prism 5.0 (GraphPad Software, San Diego, Ca).

## Results

### Patients

The number of patients enrolled in the study and dropouts occurring at each time point are shown in Additional file 1: Table S1. Reasons for dropouts included: adverse events, non-compliance, disease worsening, therapy switch, consent withdrawal, investigator decision, and loss to follow-up. At the end of the study, the dropout rate was 42%.

The potential bias determined by the presence of anti-IFN-β neutralizing antibodies, which would impair the IFN-β signaling cascade and biological effects, was bypassed including only biologically responsive to IFN-β patients, having MxA values (NR) higher than the previously determined cut-off (NR < 3.78) [20] at 12 and 24 months of treatment (Additional file 2: Table S2).

### Flow cytometry analysis of nTreg and Tr1 in IFN-β-treated patients

Analysis of nTreg subsets and Tr1 was performed by using a multiparameter flow cytometry assay, according to the gating strategy presented in the Additional file 3: Figure S1. For each sample a total number of 25,000 events, gated on CD4^+^ cells, was acquired. This value ensures the compliance with the criteria for limit of detection (LOD) and lower limit of quantification (LLOQ) acceptance for rare cell populations. Furthermore, a minimum of 50 events, which are widely accepted as a standard threshold for reproducible enumeration of a cell population, was always measured for each of the nTreg subsets [22].

**Fig. 1:**
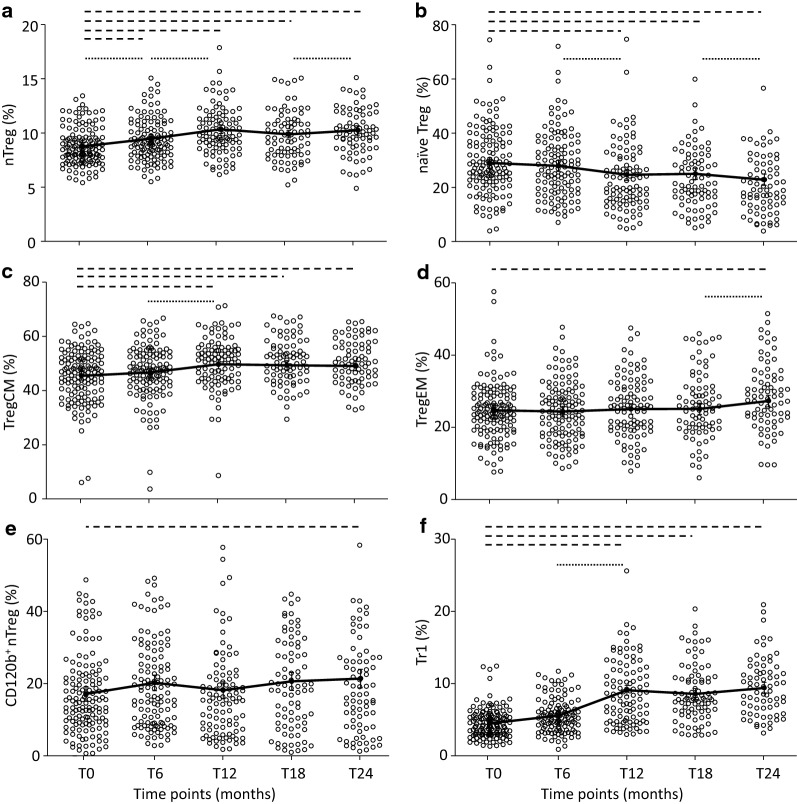
Flow cytometry quantification of nTreg and Tr1 in IFN-β-treated patients. Modulation of nTreg (**a**), naïve Treg (**b**), TregCM (**c**), TregEM (**d**), CD120b^+^ nTreg (**e**), and Tr1 (**f**) after IFN-β treatment was investigated at T0: before therapy initiation and T6, T12, T18, and T24: after 6, 12, 18, and 24 months of IFN-β, respectively. Estimated means are shown, connected by black continuous line, along with error bars representing the 95% confidence interval. Other lines indicate statistical significance (always p < 0.05): long dash lines designate differences between consecutive time points and the pre-therapy point; dotted lines designate differences between previous time points among patients

The percentage of nTreg significantly increased during the study period (Fig. 1a) compared with the percentage of nTreg at pre-therapy time point (T0); in particular, these cells significantly raised between T0 and T6, T6 and T12, and T18, and T24. The modification of nTreg percentage was characterized by the reduction of naïve Treg (Fig. 1b) and by the expansion of both TregCM (Fig. 1c) and TregEM (Fig. 1d) subsets. Indeed, the percentage of TregCM was higher than at pre-treatment period at all time points, but this increase was more evident between T6 and T12. TregEM augmented only at T24, when the number of TregEM was significantly higher in comparison to previous time points and to pre-therapy. The percentage of CD120b^+^ nTreg increased slightly, yet significantly, at T24 in comparison to pre-therapy values (Fig. 1e). The percentage of Tr1 significantly enhanced at T12 and continued to be high at the subsequent time points (Fig. 1f). In addition, despite the significant, profound decrease of the number of lymphocytes (Additional file 4: Figure S2A), the absolute number of this Treg subset increased during the therapy (Additional file 4: Figure S2F).

### Tr1-like cells after in vitro stimulation with anti-CD3/CD46 MoAbs

**Fig. 2:**
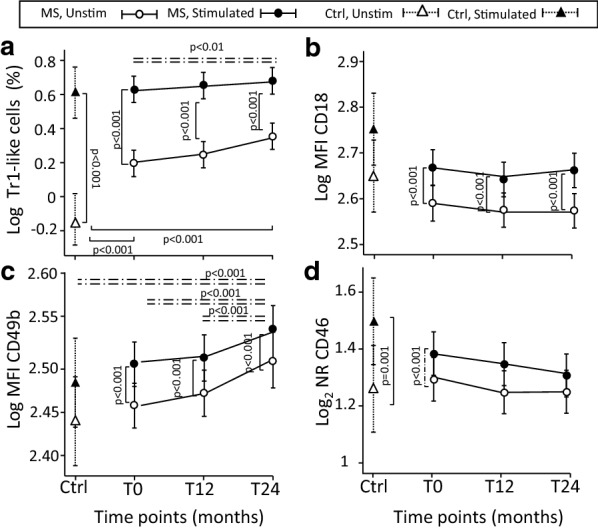
Tr1-like cell induction after in vitro anti-CD3/CD46 MoAb stimulation. Mean percentage of in vitro induced Tr1-like cells (**a**) and MFI mean of CD18 (**b**) and CD49b (**c**) markers analyzed by flow cytometry in CD4^+^ cells in IFN-β-treated patients obtained before therapy initiation (T0) and then after 12 (T12) and 24 (T24) months of therapy, and in healthy controls (Ctrl). Cells were stimulated with anti-CD3 and anti-CD46 MoAbs. mRNA levels of CD46 (**d**) in CD4^+^ cells pre- and post- in vitro stimulation. Results are calculated as normalization ratio (NR), relative to a common standard sample. Estimated means are shown, connected by continuous lines, along with error bars representing the 95% confidence interval. Other lines indicate statistical significance

**Fig. 3:**
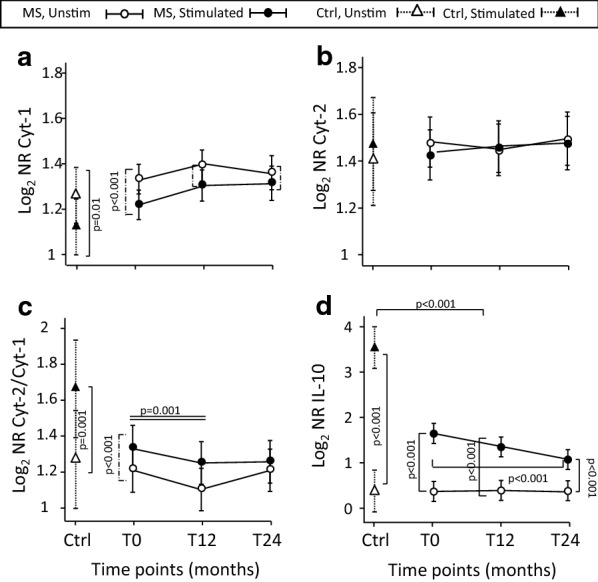
Cyt-1, Cyt-2, and IL-10 mRNA quantification after in vitro anti-CD3/CD46 MoAb stimulation. mRNA levels of Cyt-1 (**a)**, Cyt-2 (**b**), Cyt-2 and Cyt-1 ratio (**c**), and IL-10 mRNA levels (**d**) in CD4^+^ cells pre- and post- in vitro stimulation. CD4^+^ cells obtained from IFN-β-treated patients before therapy initiation (T0) and then after 12 (T12) and 24 (T24) months of therapy and healthy controls (Ctrl) and stimulated with anti-CD3 and anti-CD46 MoAbs. Results are calculated as normalization ratio (NR), relative to a common standard sample. Estimated means are shown, connected by continuous lines, along with error bars representing the 95% confidence interval. Other lines indicate statistical significance

The percentage of Tr1-like cells (defined by the CD4^+^CD18^+^CD49b^+^ phenotype) significantly increased after in vitro stimulation with anti-CD3 and anti-CD46 MoAbs (ANOVA main effect p < 0.001; interaction not significant) in both patients and Ctrl. In addition, in patients, IFN-β induced a slightly significant increase in Tr1-like cells at T24 compared to pre-therapy period (Fig. 2a). After stimulation, the expression of the CD18 surface marker, determined by flow cytometry as MFI, only increased (ANOVA main effect p < 0.001; interaction not significant) in patients (for Ctrl, p = 0.07, not significant), but was not affected by IFN-β therapy, because it remained unchanged over time in treated patients (Fig. 2b). Conversely, the expression of CD49b seems to be affected by the long term therapy because not only its MFI increased after stimulation, but it also raised independently of the in vitro stimulation (i.e. in both unstimulated and stimulated cells; ANOVA interaction term not significant) (Fig. 2c). CD46 pathway markers were studied before and after stimulation by determining the levels of mRNA for CD46 molecule and its two isoforms, Cyt-1 and Cyt-2. In both untreated patients and Ctrl, the anti-CD3/CD46 MoAb stimulation induced the expression of CD46 mRNA (Fig. 2d), but this effect was unrelated to therapy (ANOVA interaction between “time and stimulation” not significant and “time effect” not significant). However, the levels of mRNA for Cyt-1 isoform decreased after anti-CD3/CD46 MoAb stimulation in patients untreated (at T0) and treated (at T12 and T24) and in Ctrl (Fig. 3a). On the contrary, the stimulation had no modulation effect on Cyt-2 mRNA, which levels were significantly higher than those of Cyt-1 (p < 0.001 at all time points) in patients and in Ctrl (Fig. 3b). This pattern was confirmed by the high and significant correlation, present at all time points and stimulation conditions, between Cyt-1 and Cyt-2 levels (Additional file 5: Figure S3). The ratio of Cyt-2 to Cyt-1 mRNA levels increased after stimulation in both patients and Ctrl (Fig. 3c), and this is in line with previous results indicating that the two isoforms have opposite effects (Cyt-1, immunosuppressive, and Cyt-2, immunostimulating) [12]. The relative reduction of Cyt-1 mRNA in stimulated cells (denominator) with a stable Cyt-2 mRNA (numerator) determined the increased ratio. The mean ratio was significantly reduced at 12 months of IFN-β treatment both in stimulated and unstimulated CD4^+^ cells (ANOVA interaction not significant) suggesting that IFN-β may alter the relative expression of the two isoforms in favour of Cyt-1 (relative decrease in Cyt-2, numerator, and/or increase in Cyt-1, denominator).

Unstimulated CD4^+^ cells from patients, at all three time points, and from Ctrl contained very low levels of IL-10 mRNA (Fig. 3d). This cytokine can be produced by Tr1 and is considered important for modulating T-cell response. After in vitro induction of Tr1-like cells, IL-10 levels increased in all groups of samples tested. However, IL-10 mRNA significantly diminished at T24 compared with T0 in stimulated cells obtained from IFN-β-treated patients (interaction significant). Then, we investigated, by a multivariable regression, to which extent CD18, CD49b, CD46, Cyt-1, and Cyt-2 biomarkers contributed to regulating IL-10 mRNA levels. This analysis predicted that IL-10 mRNA levels increased with CD49b levels in both stimulated and unstimulated cells and, surprisingly, with Cyt-2 mRNA expression, but only in stimulated cells (Table 1). In more detail, for every doubling of CD49b levels, the model predicted a 39% increase in IL-10 mRNA in unstimulated and a 110% increase in stimulated cells, whereas for a doubling in Cyt-2 levels, the model predicted a 47% increase in stimulated cells. By contrast, the effect of Cyt-1 change on IL-10 mRNA levels was opposite because every doubling of Cyt-1 expression determined an 84% decrease in IL-10 mRNA in stimulated cells.

**Table 1:** Multivariable regression analysis of predicting factors affecting IL-10 mRNA levels

	Effect on log_2_ IL-10	95% confidence interval	p*	Effect on IL-10	% of change**	95% confidence interval
log_2_ CD49b in:						
Unstimulated	0.476	0.157: 0.796	*0.003*	*1.39*	+ 39	1.115: 1.736
Stimulated	1.068	0.769: 1.367	*0.000*	*2.1*	+ 110	1.704: 2.580
log_2_ Cyt-1 in:						
Unstimulated	− 0.097	− 0.795: 0.601	0.785	*0.93*	− 7	0.576: 1.517
Stimulated	− 1.961	− 2.562: − 1.360	*0.000*	*0.26*	− 84	0.169: 0.390
log_2_ Cyt-2 in:						
Unstimulated	0.067	− 0.432: 0.565	0.793	*1.05*	+ 5	0.741: 1.479
Stimulated	0.558	0.121: 0.995	*0.012*	*1.47*	+ 47	1.088: 1.992
Stimulated vs unstimulated at:						
T0	2.275	1.457: 3.093	*0.000*	*4.84*	+ 384	2.746: 8.534
T12	2.107	1.254: 2.960	*0.000*	*4.31*	+ 331	2.384: 7.782
T24	1.822	0.976: 2.667	*0.000*	*3.53*	+ 253	1.967: 6.352

*Calculated by exponentiating the coefficients reported in “effect on log_2_ IL-10 column”

**Representing the % of change in IL-10 mRNA levels per each hypothetical doubling of the respective predictor level

### Association between Treg levels and disease activity

Due to profound inter-individual variations in the modulation of TregCM, TregEM, CD120b^+^ nTreg, and Tr1 subsets, patients were divided into three groups based on the variations of each Treg subset (increase, maintenance or a slight increase, or decrease) at 24 months of therapy. We found no evidences of EDSS changes (at most a half point change) in the different groups (Additional file 6: Figure S4).

Patients were also divided into two groups based on the presence of clinical relapses or a stable course of the disease (Table 2).

**Table 2:** Baseline characteristics of patients who experienced or not clinical relapses during the study course

	Relapse yes (n = 31)	Relapse no (n = 117)	Significance
Gender (♀); patients (%)	20 (64.51)	72 (61.53)	χ^2^ = 0.92, p = 0.761
Age (years); mean (SD)	30.45 (± 7.66)	38.02 (± 10.42)	*t *= − *3.77, p *<* 0.001*
EDSS*; mean (SD)	1.32 (± 0.83)	1.55 (± 0.86)	t = − 1.34, p = 0.181
Enhancing lesions**; patients (%)	18/31 (58)	55/81 (67.9)	χ^2^ = 1.36, p = 0.243
Naïve Treg percentage*; mean (SD)	33 (± 14)	28.16 (± 11.09)	*t *=* 2.03, p *=* 0.044*
TregCM percentage*; mean (SD)	42.63 (± 13.12)	46.52 (± 8.17)	*t *=* − 2.04, p *=* 0.043*
TregEM percentage*; mean (SD)	23.39 (± 6.28)	25.06 (± 8.10)	t = 0.27, p = 0.290
Tr1 percentage*; mean (SD)	3.83 (± 1.22)	4.68 (± 2.13)	*t *=−* 2.10, p *=* 0.037*

*SD* standard deviation, *Treg* regulatory T cells, *TregCM* Treg central memory, *TregEM* Treg effector memory

p-value calculation was done using Chi squared (χ^2^) or two independent sample t-test for categorical or continuous variables, respectively

* Data determined at the pre-therapy time point

** These data are available for a subgroup of patients only

Compared with stable patients, those experiencing relapses were younger, had a higher percentage of naïve Treg and a lower percentage of TregCM and Tr1 at pre-therapy time point. A mixed ANOVA on the percentages of each Treg subsets, performed with relapses (two levels: yes/no) and time (5 levels: T0, T6, T12, T18, and T24 time points) as independent variables, and age at pre-therapy as covariate, revealed no differences in Treg subset percentages in patients with or without experience of clinical relapses. As the main effect of IFN-β on Treg values was most evident in the first months of treatment, analyses were repeated considering only T0, T6, and T12 time points and comparing patients who had relapses in the first year of therapy vs those without relapses, but the results did not change. However, since this last analysis highlighted a trend toward significance for TregCM (group x time interaction (F[2200] = 1.49, p = 0.08), a Newman Keuls post hoc test was performed, revealing an increment over time of TregCM percentage (T0 vs T12 and T6 vs T12: p = 0.001) in the group with stable disease.

Patients were also divided into two groups depending on whether they manifested or not new lesions in T2 or new enhancing lesions at MRI (Additional file 7: Table S3). This analysis was performed only in patients in whom MRI and experimental data of all Treg subsets, at all time points, were available. Compared with radiologically stable patients, those with new lesions in T2 were younger and had more enhancing lesions at pre-therapy. Then, a mixed ANOVA on each Treg subsets was performed using new lesions (two levels: yes/no) and time (5 levels: T0, T6, T12, T18, and T24 time points) as independent variables, and age at pre-therapy as a covariate. The critical interaction “new lesions x time” was not significant, indicating that the longitudinal changes of Treg subsets never differed between the two groups. This result remained unchanged in patients experiencing new lesions in the first year of therapy compared with those without new lesions. However, the mixed ANOVAs on the percentage of Tr1 demonstrated a statistically significant critical interaction “group x time” (F[4228] = 2.51, p = 0.04) and Newman Keuls post hoc test highlighted that, in the group of patients stable at MRI, Tr1 values grew at every time points. In patients with reactivation at MRI there was no Tr1 increase.

## Discussion

The analysis of Treg subsets performed in naïve and IFN-β-treated patients led to conflicting results [9–14, 19], possibly due to differences in study design, type of enrolled patients, sample size, timing of treatment, time between disease onset and treatment inception, duration of follow-up, type and dosage of IFN-β, and approaches used for identifying and analyzing Treg. The current study, that overcomes some of the limitations of the previous works, demonstrated that specific Treg subsets gradually increased at 6 months of IFN-β treatment and continued to augment during the whole treatment period. This result is in congruence with previous studies reporting that the suppressive function and frequency of peripheral nTreg have been improved in the first months of therapy [9–11]. However, we also observed that IFN-β therapy induced an increased percentage of memory Treg subsets, although the observed expansion undertook at different time points for each subset. TregCM firstly expanded (between T6 and T12), while TregEM only increased later (between T18 and T24). Of note, TregCM were the most effective nTreg cells, due to the expression of CCR7. The expansion of memory Treg was counterbalanced by a reduction of naïve Treg. The percentage of Tr1 cells significantly increased from T6 and T12 and remained high for the entire observation period. Despite the profound, significant decrease of the number of lymphocytes, a well-known effect of IFN-β therapy [23], also the absolute number of Tr1 increased during the therapy.

These findings suggest that IFN-β induces coordinated actions in different Treg subsets by both inhibitory and stimulatory feedbacks leading to a balanced response that fulfils its protective function while preventing immunopathology.

As reported for several other immune cells [24], we also found that Treg subsets from patients vary widely before and after therapy, which may indicate the use of adaptive strategies, compensatory pathways and functional redundancy to maintain their functions.

The comparison of patients according to presence or absence of relapses revealed that clinically stable patients displayed an increase in TregCM and a decrease in naïve Treg over time. This data is consistent with higher suppressive activity of TregCM subset and low suppressive action of naïve Treg [25]. In addition, Tr1 constantly increased only in patients with stable MRI while in those with new lesions the percentage of Tr1 increased in the first year of therapy, but then decreased.

A modulation of Tr1 was observed in in vitro experiments. Indeed, treated patients showed higher CD49b mRNA expression suggesting that this molecule might function as a marker associated with the therapeutic effects of IFN-β. Conversely, CD18 mRNA was not associated with the outcome of IFN-β therapy. Accordingly, CD18 is known to be a less specific marker since it is expressed by many cell types [26].

We also assessed the involvement of the CD46 pathway in determining the induction of the Tr1-like phenotype in CD4^+^ cells from patients. Despite the ex vivo CD18^+^CD49b^+^ Tr1 percentage increase, the suppressive function of Tr1 appeared to be defective in patients since the levels of IL-10 mRNA, produced after in vitro stimulation by Tr1-like cells, were lower than those observed in healthy subjects. In addition, IL-10 mRNA expression further decreased in patients treated for 24 months with IFN-β. These results are aligned with those reporting that, under similar in vitro conditions, cells from IFN-β-treated and untreated patients secreted less IL-10 (measured by an ELISA assay) than cells from Ctrl [27]. In addition, anti-CD3/CD46-stimulated CD4^+^ cells of patients exhibited a defective response to IL-10 stimulation not reverted by IFN-β treatment [13]. Therefore, our in vitro results indicate that, in patients, CD3/CD46-induced Tr1-like cells are only partially functioning and IFN-β might not be fully capable of restoring this impairment. Furthermore, these data also suggest that CD18^+^CD49b^+^ Tr1 cells identified ex vivo after IFN-β treatment may be different from the Tr1-like cells induced by anti-CD3/CD46 MoAb stimulation and may exert different activities Indeed, it has been proposed that Treg adopt several different mechanisms for the down modulation of immune responses. One of these mechanisms, the ectoenzymatic activity, can be particularly relevant in CNS autoimmunity [28]. In addition, Treg may also show other functions, not limited to immune suppression. For instance, these cells are able to promote oligodendrocyte differentiation and remyelination in a mouse model of lysolecithin-induced focal demyelination. This occurs through the release of CCN3, a growth-regulatory protein usually expressed in the developing brain [29].

Dropouts were high in this study, but this does not weaken the conclusions because the modulations of Treg subsets we have observed at 24 months from therapy beginning were already found at 12 months of therapy, when the dropout was lower. Dropouts rates are known to differ widely according to study duration and study type (randomized controlled trial or observational). However, a 38% dropout in IFN-β 1a-treated patients, at a median of 421 days of therapy [30], and a dropout in 37% at median time of 1.7 years in a recent real-life clinical study [31], have been recently reported. Finally, patients treated with subcutaneous IFN-β-1a have a probability of persistence on therapy at 3 years of 43.6% [32]. These values are similar to that we have found in this study.

The biological basis of altered T-cell response in patients after anti-CD3/CD46 MoAb stimulation was also elucidated by quantifying the levels of mRNA for Cyt-1 and Cyt-2 isoforms. In a CD46 transgenic mouse model, Cyt-1 and Cyt-2 isoforms proved to play antagonistic roles in inflammation [12, 33], with Cyt-1 promoting the suppression of the inflammatory reaction and Cyt-2 inducing overall increase in inflammation by interfering with IL-10 production [12]. As previously described [27], we demonstrated a higher level of expression of Cyt-2 compared to Cyt-1, regardless of in vitro stimulation, with no significant differences between patients and Ctrl and no change after IFN-β treatment. However, differently to the report that described a significant increase in the CD46-induced Cyt-2 isoform, with no changes in Cyt-1 in patients, either treated or untreated with IFN-β [27], we found a decrease of Cyt-1 mRNA, due to anti-CD3/CD46 MoAb stimulation. Of note, our work overcomes the limitations of the previous one, which included reduced number of patients, different numbers of treated and untreated patients, and only one-time point for data collection. Considering that the expression of these two subunits may change during different stages of T-cell activation [12], and that they may be reciprocally regulated, we also calculated the ratio of Cyt-2-to-Cyt-1 mRNA expression. We found a slight, yet significant, decrease in Cyt-2-to-Cyt-1 ratio after IFN-β treatment compared to pre-treatment values, although the same ratio increased at all time points upon anti-CD3/CD46 MoAb stimulation both in patients and Ctrl when compared to the unstimulated counterpart. To better evaluate the reciprocal role of Cyt-1 and Cyt-2 in influencing IL-10 mRNA levels and elucidate the reasons for the minor induction of IL-10 we fitted a multivariable regression. This analysis reported a strong positive association between the expression of the Tr1-associated gene CD49b and IL-10 but, at the same time, unexpectedly, a positive association between IL-10 and Cyt-2 mRNA levels and a negative association between IL-10 and Cyt-1 mRNA levels. Once again, this finding may represent another facet of the disrupted IL-10 pathway in MS which is uncorrected by IFN-β treatment.

## Conclusion

IFN-β induces the expansion of nTreg subsets endowed with a high suppressive activity, especially in clinically stable patients (without relapses and new MRI lesions); this may represent one of the mechanisms by which the IFN-β exerts its effects. IFN-β also induced a strong expansion of Tr1 but, surprisingly, in vitro expansion of Tr1-like cells yielded a low production of IL-10, which is the typical Tr1 suppressive cytokine.

## Supplementary information

**Additional file 1: Table S1.** Number of patients included in the study and dropouts occurring at each time points. The therapy time points were: T0, before therapy initiation; T6, T12, T18 and T24, after 6, 12, 18, and 24 months of IFN-β, respectively.

**Additional file 2: Table S2.** Number of samples received at T12 and T24 for MxA quantification and patient subdivision according to MxA induction. The MxA quantification was performed before IFN-β therapy initiation (T0) and then after 12 (T12) and 24 (T24) months of therapy.

**Additional file 3: Figure S1.** The gating strategy ensures the quality and continuity of data acquired for the whole blood and allows the identification of: (A) single-cell lymphocytes, which can be distinguished by their forward scatter/side scatter properties;(B) after lymphocyte gating, doublets are excluded, followed by exclusion of dead or autofluorescent cells; (C) naive, central memory (CM), and effector memory (EM) CD4^+^ cells based on the expression of C45RA and CCR7 markers; (D) CD4^+^ Tr1 cells based on the expression of CD18 and high level of CD49b expression; (E) CD4^+^ Treg cells based on positive expression of CD25 and low/negative expression of CD127; and (F) Treg subsets population (naïve Treg, TregCM, and TregEM) based on the expression of C45RA and CCR7 markers. (G and H) CD120b (TNFR2) expressing cells (black line) within nTreg population (dark grey) compared to naïve CD4^+^ lymphocytes (grey) and monocytes (light grey), which are respectively negative and positive for the expression of CD120b antigen.

**Additional file 4: Figure S2.** The absolute number of total lymphocytes (A), naïve Treg (B), TregCM (C), TregEM (D), CD120b^+^ nTreg (E), and Tr1 (F) after IFN-β treatment was investigated at T0: before therapy initiation and T6, T12, T18, and T24: after 6, 12, 18, and 24 months of IFN-β, respectively. Estimated means are shown, connected by continuous lines, along with error bars representing the 95% confidence interval. Black lines indicate statistically significant decreases, while red lines indicate statistically significant increases.

**Additional file 5: Figure S3.** Correlation between Cyt-1 and Cyt-2 mRNA expression after stimulation. Scatterplot representing the correlations between Cyt-1 and Cyt-2 values at the indicated time points and conditions. Black lines were obtained by linear regression.

**Additional file 6: Figure S4.** Subdivision of patients according to the variations of each Treg subset at T24. Patients were grouped based on the increase (↑), maintenance or slight increase (=/↑), or decrease (↓) of the different Treg subset percentages after 24 months of IFN-β therapy. Therefore, only 86 patients that completed the follow-up were included in the analysis. TregCM ↑: ≥ 6%, =/↑: -1 to < 6%, ↓: < -1%; TregEM ↑: ≥ 5%, =/↑: -1 to < 5%, ↓: < -1%; CD120b^+^ nTreg ↑: ≥ 10%, =/↑: 0 to < 10%, ↓: < 0%; Tr1↑: ≥ 4.5%, =/↑: 0 to < 4.5%, ↓: < 0%. In red: median EDSS value.

**Additional file 7: Table S3.** Association with disease activity. Patients were divided in two groups depending on whether they manifested or not new lesions in T2 or new enhancing lesions.

## Data Availability

The dataset analyzed during the current study are available from the corresponding author on reasonable request. All data generated or analyzed during this study are included in this published article (Additional files: 1, 2, 3, 4, 5, 6, 7).

## Abbreviations

Ct: Cycle threshold
Ctrl: Controls
FCS: Fetal calf serum
IFN-β: Interferon-beta
IL-2: Interleukin-2
IL‐7R: Interleukin-7 receptor alpha chain
IL-10: Interleukin-10
iTreg: Inducible regulatory T cells
MFI: Mean fluorescence intensity
MoAbs: Monoclonal antibodies
MRI: Magnetic resonance imaging
MS: Multiple sclerosis
MxA: Mixovirus resitance protein A
NR: Normalization ratio
nTreg: Naturally regulatory T cells
PBMC: Peripheral mononuclear blood cells
RRMS: Relapsing–remitting multiple sclerosis
TNFR2: Tumor necrosis factor receptor 2
Treg: Regulatory T cells
TregCM: Central memory regulatory T cells
TregEM: Effector memory regulatory T cells
