# Supplementary figures and images for: Simultaneous quantification of natural and inducible regulatory T-cell subsets during interferon-β therapy of multiple sclerosis patients
Source: J Transl Med. 2020 Apr 16;18:169. doi: 10.1186/s12967-020-02329-5 (PMC7161224; doi:10.1186/s12967-020-02329-5)

## Slide 1
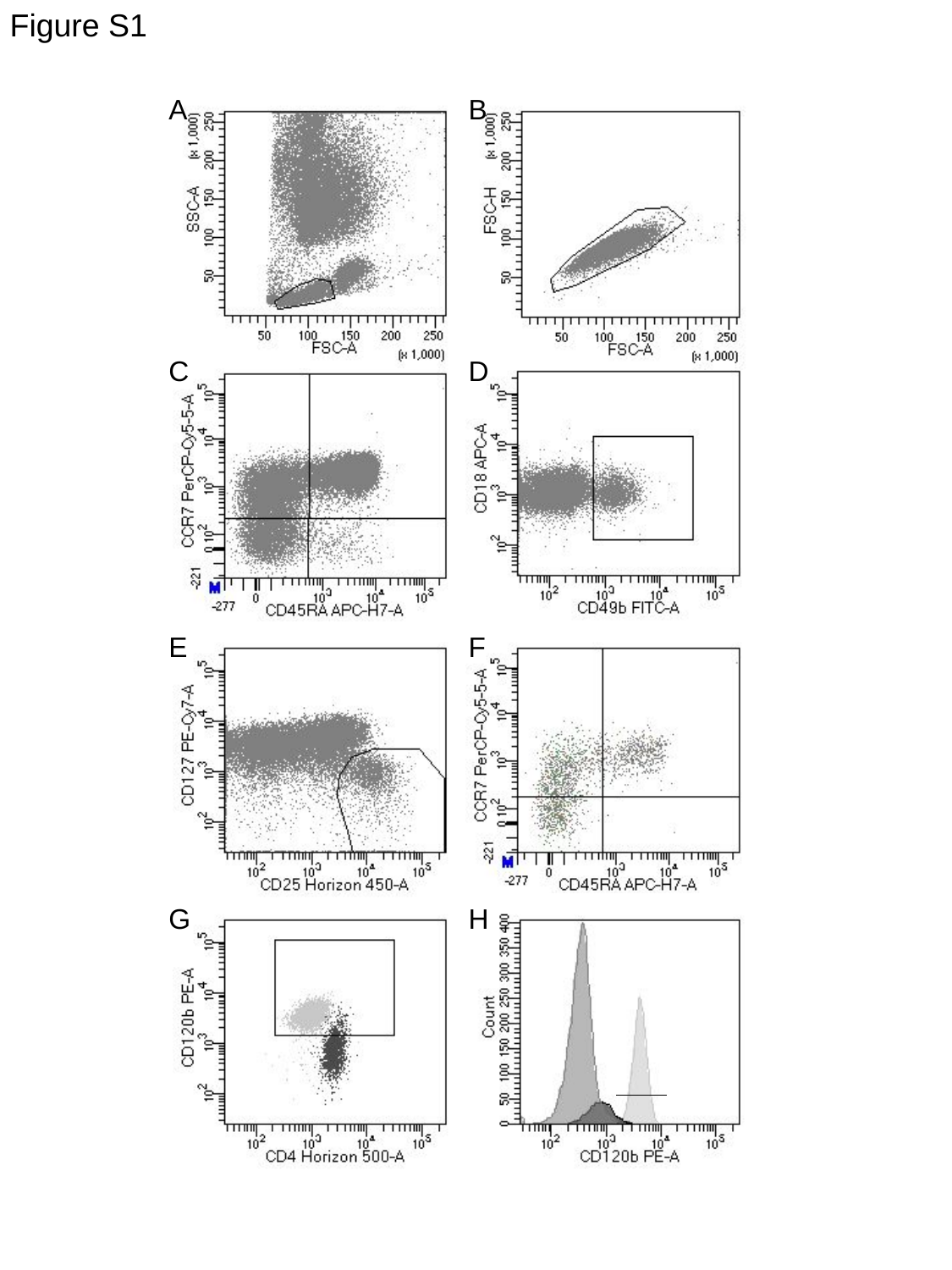

Figure S1
A
B
C
D
E
F
G
H

Supplement: Supplementary file 3 — Additional file 3: Figure S1. The gating strategy ensures the quality and continuity of data acquired for the whole blood and allows the identification of: (A) single-cell lymphocytes, which can be distinguished by their forward scatter/side scatter properties;(B) after lymphocyte gating, doublets are excluded, followed by exclusion of dead or autofluorescent cells; (C) naive, central memory (CM), and effector memory (EM) CD4+ cells based on the expression of C45RA and CCR7 markers; (D) CD4+ Tr1 cells based on the expression of CD18 and high level of CD49b expression; (E) CD4+ Treg cells based on positive expression of CD25 and low/negative expression of CD127; and (F) Treg subsets population (naïve Treg, TregCM, and TregEM) based on the expression of C45RA and CCR7 markers. (G and H) CD120b (TNFR2) expressing cells (black line) within nTreg population (dark grey) compared to naïve CD4+ lymphocytes (grey) and monocytes (light grey), which are respectively negative and positive for the expression of CD120b antigen. [file 12967_2020_2329_MOESM3_ESM.pptx]

## Slide 1
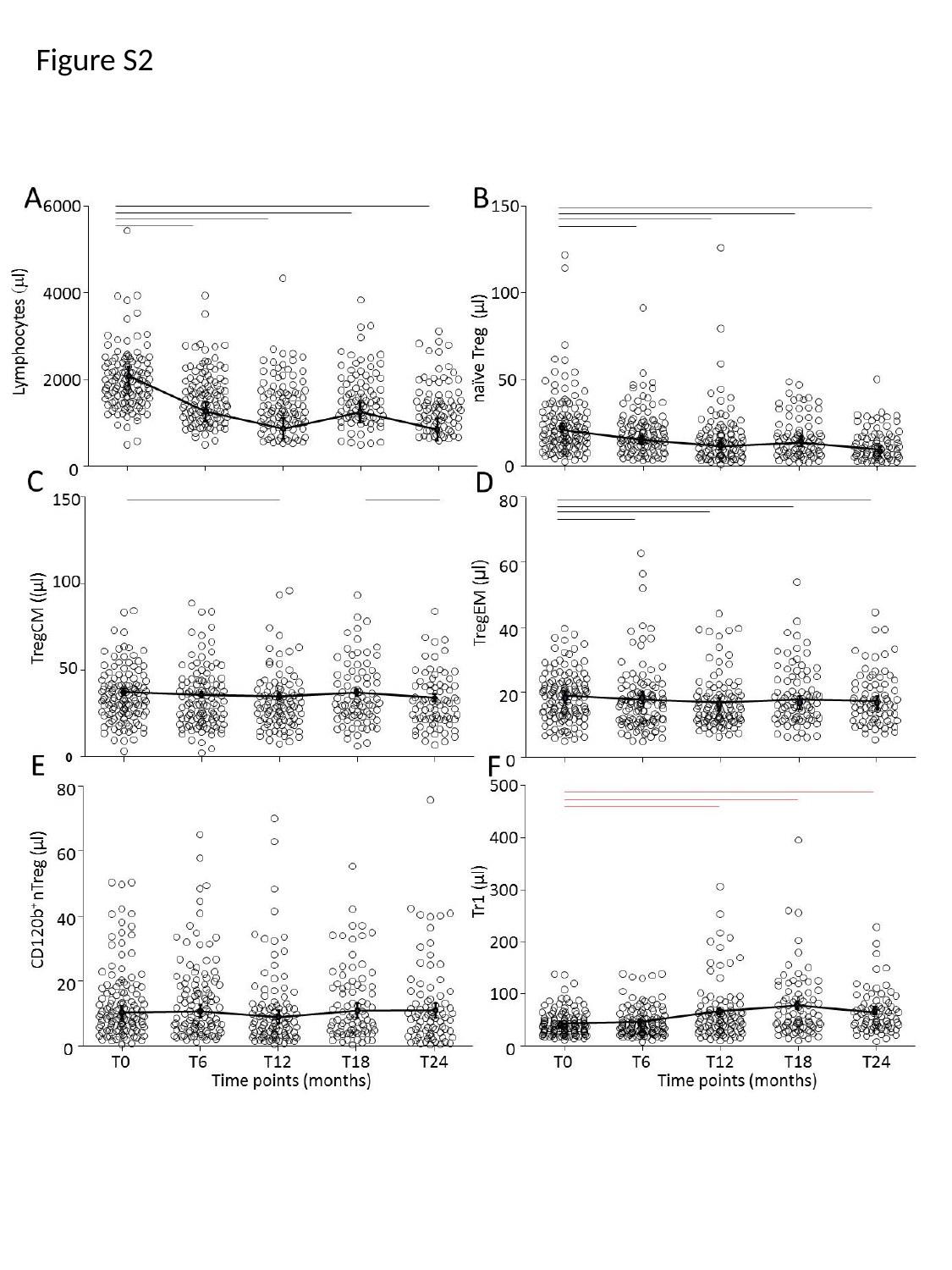

Figure S2

Supplement: Supplementary file 4 — Additional file 4: Figure S2. The absolute number of total lymphocytes (A), naïve Treg (B), TregCM (C), TregEM (D), CD120b+ nTreg (E), and Tr1 (F) after IFN-β treatment was investigated at T0: before therapy initiation and T6, T12, T18, and T24: after 6, 12, 18, and 24 months of IFN-β, respectively. Estimated means are shown, connected by continuous lines, along with error bars representing the 95% confidence interval. Black lines indicate statistically significant decreases, while red lines indicate statistically significant increases. [file 12967_2020_2329_MOESM4_ESM.pptx]

## Slide 1
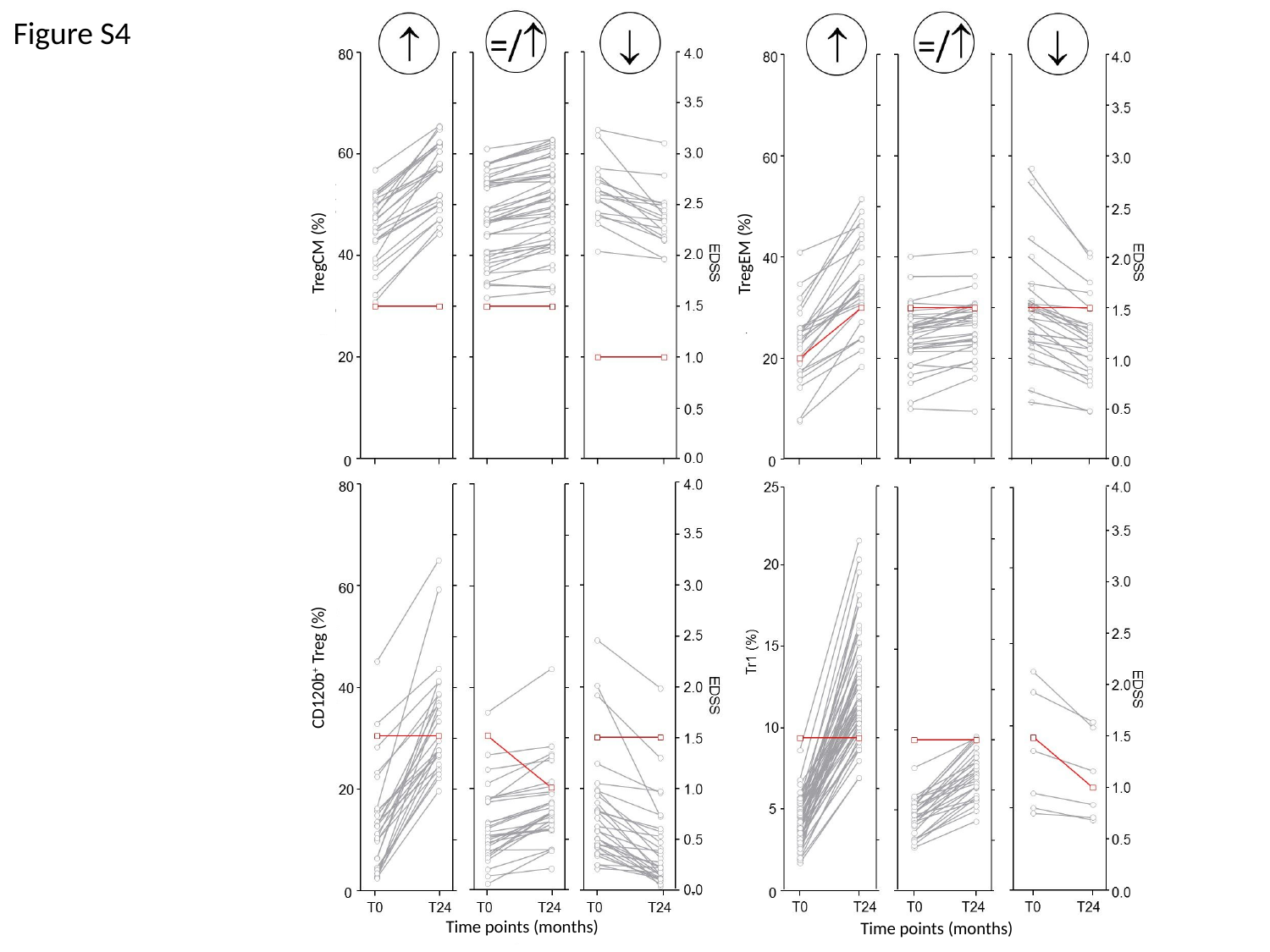

TregCM (%)
TregEM (%)
CD120b+ Treg (%)
Time points (months)
Time points (months)
Figure S4

Supplement: Supplementary file 6 — Additional file 6: Figure S4. Subdivision of patients according to the variations of each Treg subset at T24. Patients were grouped based on the increase (↑), maintenance or slight increase (=/↑), or decrease (↓) of the different Treg subset percentages after 24 months of IFN-β therapy. Therefore, only 86 patients that completed the follow-up were included in the analysis. TregCM ↑: ≥ 6%, =/↑: -1 to < 6%, ↓: < -1%; TregEM ↑: ≥ 5%, =/↑: -1 to < 5%, ↓: < -1%; CD120b+ nTreg ↑: ≥ 10%, =/↑: 0 to < 10%, ↓: < 0%; Tr1↑: ≥ 4.5%, =/↑: 0 to < 4.5%, ↓: < 0%. In red: median EDSS value. [file 12967_2020_2329_MOESM6_ESM.pptx]
