# Supplementary material for: Simultaneous quantification of natural and inducible regulatory T-cell subsets during interferon-β therapy of multiple sclerosis patients
Source: J Transl Med. 2020 Apr 16;18:169. doi: 10.1186/s12967-020-02329-5 (PMC7161224; doi:10.1186/s12967-020-02329-5)
Supplement: Supplementary file 5 — Additional file 5: Figure S3. Correlation between Cyt-1 and Cyt-2 mRNA expression after stimulation. Scatterplot representing the correlations between Cyt-1 and Cyt-2 values at the indicated time points and conditions. Black lines were obtained by linear regression. [file 12967_2020_2329_MOESM5_ESM.pptx]

## Slide 1
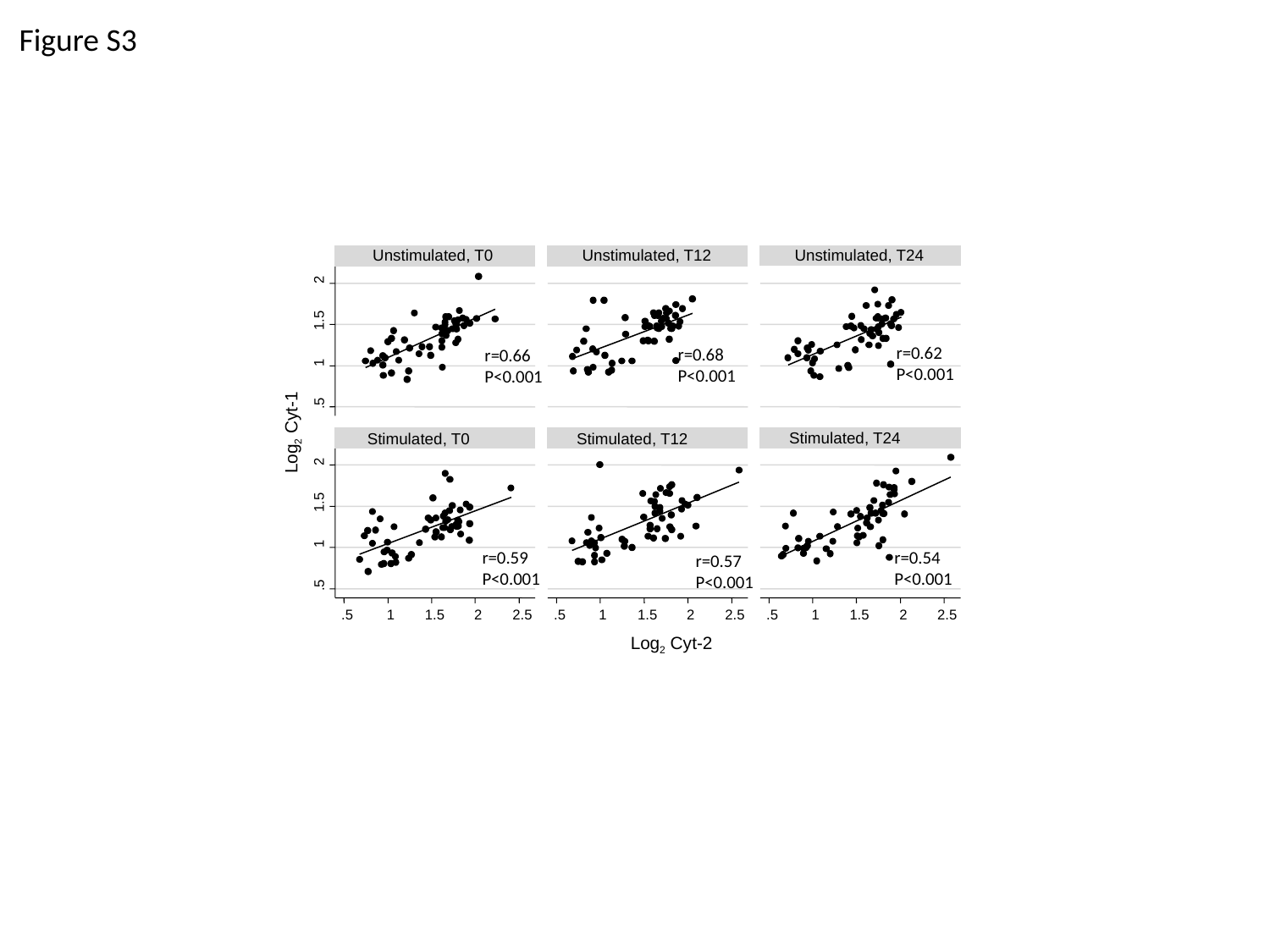

Figure S3
Unstimulated, T0
Unstimulated, T12
Unstimulated, T24
2
1.5
r=0.62
P<0.001
r=0.68
P<0.001
r=0.66
P<0.001
1
.5
Log2 Cyt-1
Stimulated, T24
Stimulated, T0
Stimulated, T12
2
1.5
1
r=0.54
P<0.001
r=0.59
P<0.001
r=0.57
P<0.001
.5
.5
1
1.5
2
2.5
.5
1
1.5
2
2.5
.5
1
1.5
2
2.5
Log2 Cyt-2
